# Sport activity in Charcot–Marie–Tooth disease: A case study of a Paralympic swimmer

**DOI:** 10.1016/j.nmd.2016.06.002

**Published:** 2016-09

**Authors:** Giuseppe Vita, Stefania La Foresta, Massimo Russo, Gian Luca Vita, Sonia Messina, Christian Lunetta, Anna Mazzeo

**Affiliations:** a Unit of Neurology, Department of Clinical and Experimental Medicine, University of Messina, Messina, Italy; b Nemo Sud Clinical Centre, Aurora Onlus Foundation, AOU Policlinico, Messina, Italy

**Keywords:** Charcot-Marie-Tooth, Sport, Swimming, Paralympic record, Quality of life, Self-esteem

## Abstract

This study reports the positive physical, emotional and psychosocial changes induced by sport activity in a Paralympic swimmer with Charcot–Marie–Tooth (CMT) type 4A. When we compared evaluations before initiating sport activity with those after five years of competitive activity, we found: i) increased proximal muscles strength of upper limbs; ii) augmented ability to propel wheelchair independently; iii) improved quality of life; iv) reduced trait anxiety and striking improvement of depression; v) enhanced self-esteem. Longitudinal studies in large cohorts to evaluate the positive effects of sport activity are needed to support provision of evidence-based advice to patients and families.

## Highlights

• The paper reports a wheelchair-bound CMT 4A patient who became a Paralympic swimmer.
• She regularly performed intensive aerobic workout and competed in sprint distance events.
• She became a backstroke and freestyle gold medalist in the Italian Championships.
• Sport activity increased proximal muscle strength of upper limbs and improved QoL.
• Sport activity reduced anxiety, annulled depression and increased self-esteem and self-efficacy.

## 1 Introduction

Sport activity is a valuable tool to improve sense of wellness, quality of life and to break down social barriers of discrimination for disabled individuals [1]. Apart from the issue of autonomy of the individual, many physical, psychological and social benefits arise from sporting participation, which can translate into reduced health-care costs [2]. Available evidence reveal also a positive impact of sports on areas of self-esteem, self-efficacy and mental health [3].

The main impairment types in Paralympic sports include amputation or limb deficiency, cerebral palsy, spinal cord-related disability, visual impairment, and intellectual impairment. A sixth group, known as *les autres*, accommodates those athletes with physical impairments who are not covered by the other groups [2]. It is commonly accepted that subjects with a single disabled body segment are those preferentially prone to a sport activity. In this context, individuals with a progressive and severe genetic neuromuscular disorder are believed able to perform sports, with the exception of those competitive and with a full-body workout.

Charcot–Marie–Tooth (CMT) disease, the most common inherited neuromuscular disorder, has a quite variable course because of genotypic and phenotypic heterogeneity. CMT type 4A is caused by mutation in the gene encoding ganglioside-induced differentiation-associated protein-1 (GDAP1) on chromosome 8q21. The recessive disorder is a severe neuropathy of childhood characterized by early age of onset, rapidly progressive distal weakness and atrophy of the limbs leading to inability to walk in late childhood or adolescence, mild sensory loss with abolished deep tendon reflexes, and frequent vocal cord paresis [4].

We report here a wheelchair-bound woman with CMT type 4A who became a Paralympic champion swimmer. Longitudinal observation allowed to demonstrate, after five years of intensive aerobic physical exercise and sprint distance swimming competitions with many wins, improved proximal muscles strength with increased ability to propel wheelchair independently, enhanced quality of life (QoL) and self-esteem, remarkable improvement of depression, reduced trait anxiety.

## 2 Case report

An adult woman was referred to our clinic for a long history of muscle weakness. She started to walk at 18 months. At three years of age, because of frequent falls, she was seen by a neuropediatrician who noticed claw hands and a steppage gait. Her gait improved after bilateral Achilles tendon lengthening. By 14 years of age she developed dysphonia. She continued to have progressively increased difficulty in walking and at 25 years of age started to walk with the help of one stick and at age 28 she became wheelchair-bound. On neurological examination, at age 31 years, she was able to walk only for 1–2 meters with bilateral support. There were dysphonia for bilateral vocal cord paresis, rare dysphagia for liquids, inability to go from supine to seated position without assistance, severe claw hands and bilateral pes cavus. The patient had muscular hypotonia, mild proximal weakness and severe distal weakness (Muscle Research Council – MRC – strength grade 4 in deltoid, biceps and triceps muscles and 0 in forearm and hand muscles; at lower limbs, grade 4 in iliopsoas, 2 in quadriceps and biceps femoris, 0 in tibialis anterior, peroneus longus and halluces extensor longus). She also displayed reduced-to-absent deep tendon reflexes and deep sensory loss at legs and feet. Barthel Index (BI), which is a widely used measure of functional disability assessing the capacity of an individual to care for him/herself and to move independently or with assistance in the activities of daily living, was 55/100, and modified BI (mBI) 60/100. Nerve conduction studies showed evidence of a severe axonal sensory-motor polyneuropathy. CMT Neuropathy Score (CMTNS) resulted 33/36. Genetic study revealed that she was homozygous for a c.173_174 insA mutation in the *GDAP1* gene, determining the introduction of a premature stop codon (p.P59AfsX3). Father, mother and the brother were heterozygous for the same mutation and had a normal neurological examination.

**Fig. 1:**
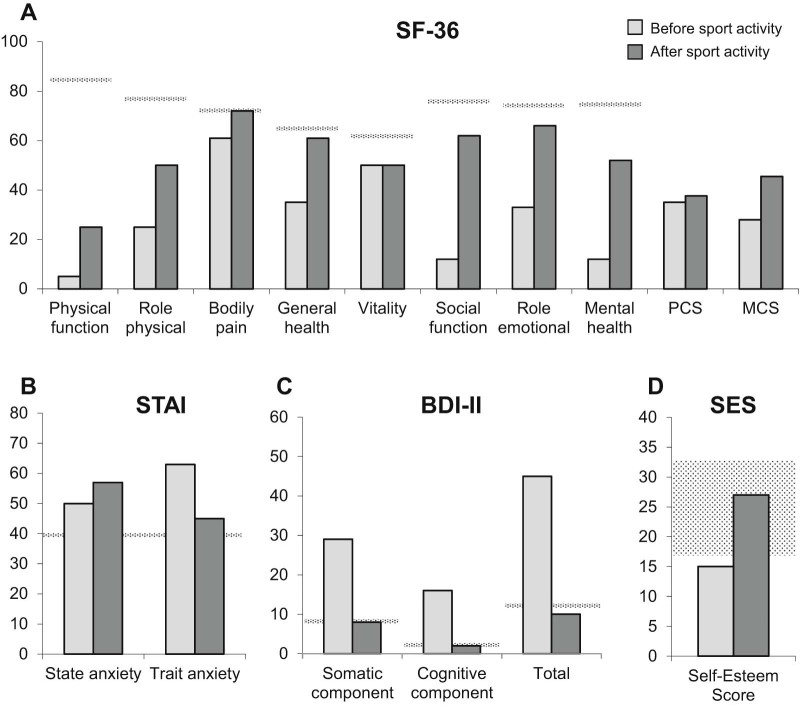
(A) SF-36 domain results before initiating sport activity and after five years of swimming activity. Pointed line indicates the mean of the Italian normative sample. (B) State-Trait Anxiety Inventory (STAI) score before and after sport activity. A higher score indicates greater anxiety, with a cut-off of 40 (pointed line). (C) Beck Depression Inventory II (BDI-II) score before and after sport activity. The pointed line indicates normal cut-off per each score. (D) Rosenberg Self-Esteem Scale (SES) score before and after sport activity. The pointed area delineates the normal self-esteem range.

On the occasion of the visit at age 31, the patient was also evaluated by a psychologist. The 36-item short-form questionnaire (SF-36) [5], [6] assessed QoL by eight specific categories of physical and emotional scores, then summarized in two scores: physical composite score (PCS) and mental composite score (MCS). Previous studies in CMT patients showed that very low scores for PCS indicate severe physical dysfunction, distressful bodily pain, frequent tiredness and unfavorable evaluation of health status. Very low scores for MCS indicate frequent psychological distress and severe social and role disability due to emotional problems [7], [8]. Anxiety was evaluated by State-Trait Anxiety Inventory (STAI) [9]. Beck Depression Inventory II (BDI-II) was used to evaluate depression symptoms [10]. Rosenberg Self-Esteem Scale (RES) measured global self-worth by evaluating both positive and negative feelings about the self [11]. All SF-36 QoL domains were markedly deteriorated with respect to the Italian normative sample, especially physical function, role physical, social function and mental health (Fig. 1). There were also high scores of state and trait anxiety, high level of somatic and cognitive elements of depression, and low self-esteem (Fig. 1).

**Fig. 2:**
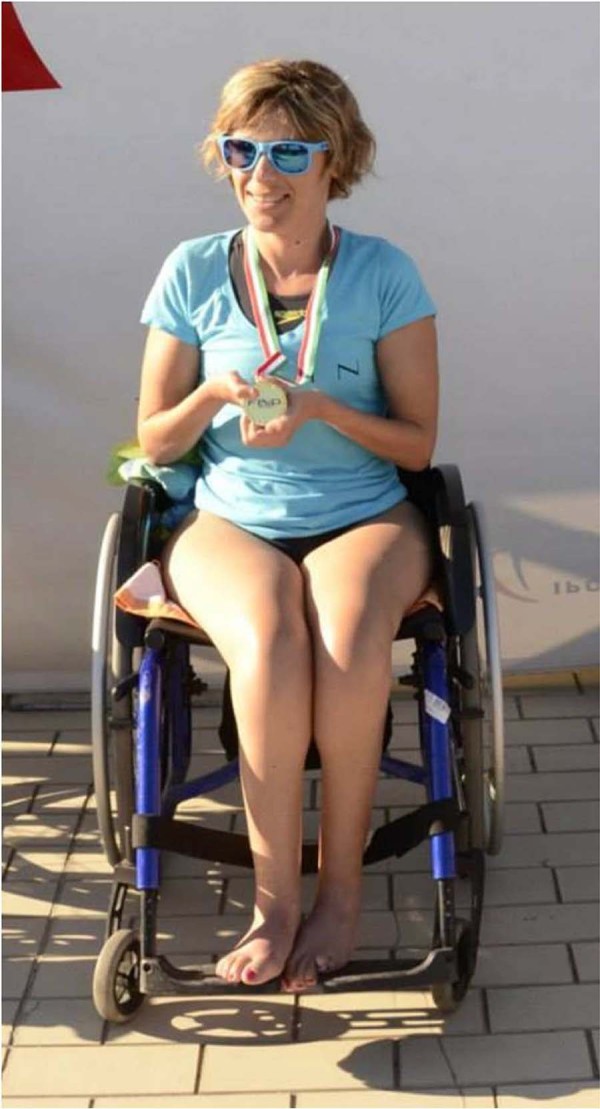
The patient at the award ceremony, Italian Championships of Paralympic Swimming, 2014, Bari. Claw hands and marked muscular wasting of forearms, hands and legs are evident.

We suggested the patient to perform physical activity. At that time, she was not able to swim, but at age 32, she was persuaded by a close friend to attend a swimming pool. She started to develop a real passion for swimming and progressively increased her workout from 25–50 m to 1200–1500 m in each pool session of approximately 1.5 hour duration, four times a week. In addition, physical training included two to three sessions of weight and aerobic exercise per week, of 90 minutes duration each, in a gymnasium. She started to play in national Paralympic competitions. As a swimmer with severe physical disability, she was classified in S3 category (range 1–10, with 1 corresponding to the most severe type of disability). In 2013 she gained the following positions at Italian Paralympic Games: silver medal, 50 m backstroke in winter; gold medal, 50 m backstroke (Italian record) and silver medal, 50 m backstroke (category record) in summer. In 2014: gold medal, 50 m backstroke; gold medal, 50 m freestyle in winter; gold medal, 100 m backstroke in summer. In 2015: gold medal, 50 m freestyle; bronze medal, 50 m backstroke, in winter (Fig. 2). Moreover, in 2014 she arrived 4th in 50 m backstroke at the International Championships in Berlin, Germany.

In 2015, at age 36, after 5 years of intensive sport activity, she had a follow-up visit in our clinic. We noticed an increased muscle strength in deltoid, biceps and triceps bilaterally (MRC grade 5-), whereas biceps femoris muscle strength decreased to grade 1. CMTNS diminished to 31/36 with reduced score from 4 to 3 in the items “Motor symptoms (arms)” and Strength (arms)”. BI was unchanged (55/100) but mBI increased to 64/100 with improvement in the wheelchair ambulation (from almost total dependence from others except short distance on flat surface to ability to propel wheelchair independently at least 50 meters).

Psychological evaluation revealed an improvement of all SF-36 domains except for a stable vitality (Fig. 1). State anxiety, as transitory emotional state, slightly increased but trait anxiety, including feelings of apprehension, tension and worry as stable personality trait during daily living activities, decreased almost reaching the normal cut-off. BDI–II showed quite decreased levels of pessimism, past failures, punishment and guilt feelings, self-dislike and worthlessness with presence of ups and downs. Self-esteem returned to normal range (Fig. 1).

Finally, the revised form of Behavioural Regulation in Exercise Questionnaire (BREQ-2) [12] demonstrated a strong self-determination as a result of high intrinsic and identified motivation regulating exercise behavior. Despite her physical problems, she exhibited strong self-determined motivation, which was suitable for her to engage in high levels of physical activity. The patient perceived increased self-esteem and self-efficacy as a consequence of sport events, which were experienced as supporting her autonomy and promoting her competence.

## 3 Discussion

A recent systematic review to evaluate benefits and risks of exercise in CMT showed that the optimal exercise modality and intensity as well as the long-term safety of exercise still remain unclear [13]. However, it appears that exercise in CMT patients may be effective in improving some components of health and fitness without harmful effects in the short-term. The majority of published studies investigated resistance training interventions, which were found to result in positive modifications in strength, functional activities, and muscle fiber size. Similarly, aerobic training led to favorable changes in some measures of strength and functional activities, as well as an increase in aerobic capacity. Combined exercise intervention studies found positive changes in ankle flexibility, balance, agility, and mobility [13]. Respiratory function has been rarely investigated in CMT patients and found minimally abnormal compared to healthy subjects, and with no amelioration after combined rehabilitation treatment [14]. Conversely, neuromuscular recovery after a fatiguing task has been found to be impaired in the vastus lateralis muscle, but not in the biceps brachii muscle, of functionally independent CMT type 1A patients, compared with healthy individuals [15]. This difference was thought due to a prevalent involvement of the lower limbs.

There are concerns that exercise may cause overwork weakness (OW), characterized by a progressive muscular weakening due to exercise, work, or daily activities in people with CMT disease, and this topic is the subject of ongoing debate with contradictory results. We have tested the OW hypothesis in 271 CMT1A patients recruited in the Italian/UK multicenter trial of ascorbic acid and did not find effect of OW over time resulting in greater weakness in dominant muscles with increasing age or in more severely affected patients [16]. The main consequence of these results is that exercise is not harmful for CMT1A patients, and possibly for the overall CMT population. Since a detrimental effect of supramaximal exercise cannot be excluded, most authors encourage physical activity in CMT patients, but recommend aerobic exercises at a submaximal work level [14], [17], [18]. However, the best training to adopt (e.g. endurance? explosive strength?) by CMT patients is not known. By definition, muscular endurance refers to the ability to perform a specific aerobic muscular action for a prolonged period of time, whereas explosive strength refers to the ability to exert strength or force as rapidly as possible in a given action with a short, very high intensity anaerobic exercise [19]. A recent study on two novel outcome measures for CMT disease, the 6-minute walk test and StepWatch™ Activity Monitor (SAM), which is an activity measuring accelerometer, showed that several SAM outputs, all reflecting the higher speed, were significantly related to the main score of physical aspect of QoL: the higher the explosive performance, the better the physical QoL [20].

Although it is an anecdotal observation, the present case study leads to two main comments. First, neuromuscular experts have not so far directed too much attention to disability sport. Based on available literature, very rarely they suggest patients to perform sport activity and, when they do, they recommend avoiding supramaximal exercise. Our patient, severely affected by CMT, not only was able to carry out regularly intensive aerobic swimming workout with progressive increase of covered distance, but she also competed in sprint distance events, in which anaerobic activity is prevalent to endurance, with many wins in national championships. After five years of sport activity, deltoid, biceps and triceps muscle strength increased from MRC grade 4 to 5- with improved mBI and CMTNS and increased ability to propel her wheelchair independently. It might be related to strengthening of deconditioned muscles which were not severely affected. The polymorphic disabilities involving people with CMT disease may lead to deconditioning and a lower tolerance for physical activities. Even a short physical performance battery is an efficient and safe tool in producing some improvement in proximal limb muscles [13], [14], [21].

The second conclusion of our report is that such an intensive muscular training induced a marked improvement of QoL, removal of depression, and reduced trait anxiety. Thanks to sport practice, the patient experienced increased self-esteem and self-efficacy leading to interpret existing situations as more autonomy-promoting and to organize her actions on the basis of personal goals and interests rather than controls and constraints [22]. A 2-year prospective study of QoL in CMT1A showed no worsening of QoL despite worsening in muscle strength and sensory function, most likely due to development of compensatory strategies that help patients cope with the slow progression of the disease [23]. We postulate that coping strategies together with the beneficial and gratifying effects of sport caused a considerable improvement of mental, emotional and psycho-social health in our patient.

Sport activity may be considered a complementary therapy of CMT, despite the present lack of proven efficacy. Longitudinal studies of its effects are needed to confirm our experience and to support provision of evidence-based advice to patients and families. Continued involvement in physical activity for both functionally independent and dependent CMT patients should be supported by clinicians.
